# A Pilot Randomized Controlled Trial of a Technology-Based Approach for Preventing Excess Weight Gain during Pregnancy among Women with Overweight

**DOI:** 10.3389/fnut.2017.00057

**Published:** 2017-11-22

**Authors:** Ariana M. Chao, Sindhu K. Srinivas, Stacia K. Studt, Lisa K. Diewald, David B. Sarwer, Kelly C. Allison

**Affiliations:** ^1^Department of Biobehavioral Health Sciences, University of Pennsylvania School of Nursing, Philadelphia, PA, United States; ^2^Department of Psychiatry, Perelman School of Medicine at the University of Pennsylvania, Philadelphia, PA, United States; ^3^Department of Obstetrics and Gynecology, Perelman School of Medicine at the University of Pennsylvania, Philadelphia, PA, United States; ^4^New York City Department of Health and Mental Hygiene, New York, NY, United States; ^5^Villanova University College of Nursing, Villanova, PA, United States; ^6^Center for Obesity Research and Education, Department of Social and Behavioral Science, Temple University College of Public Health, Philadelphia, PA, United States

**Keywords:** pregnancy, gestation, overweight, obesity, technology

## Abstract

**Objective:**

Overweight/obesity and excess weight gain during pregnancy are associated with adverse maternal and neonatal outcomes. Few interventions have been effective in limiting gestational weight gain among women with overweight or obesity. This pilot, randomized clinical trial compared treatment as usual (TAU) to a lifestyle modification program delivered *via* phone for the prevention of excess gestational weight gain in women who had overweight or obesity.

**Methods:**

Participants included 41 pregnant women with a body mass index (BMI) ≥ 25 kg/m^2^ (mean age = 28.7 ± 5.8 years; mean pre-gravid BMI = 31.2 ± 6.2 kg/m^2^; 54% black, 39% white). The intervention group (*n* = 20) received weekly telephone counseling sessions and used WiFi scales to monitor their weight from weeks 16 to 36 of pregnancy. We compared differences in weight and birth outcomes for the intervention vs. the TAU group (*n* = 21).

**Results:**

The intervention and TAU groups did not differ with respect to: gestational weight gain (15.5 ± 5.3 vs. 13.3 ± 6.8 kg, respectively); proportion gaining above the 2009 Institute of Medicine recommended weight range (83 vs. 70%); and weight gain from pre-pregnancy weight to 6 weeks postpartum (4.8 ± 4.6 vs. 3.0 ± 5.5 kg). Other birth and health outcomes also did not differ.

**Conclusion:**

A telemedicine intervention designed to decrease logistical burden on participants was not more successful in reducing excessive weight gain during pregnancy as compared to TAU. Future studies should examine more intensive forms of remote treatment beginning earlier in pregnancy as well as interventions promoting a healthy weight prior to pregnancy.

## Introduction

Preventing excess gestational weight gain is one of the most significant modifiable risk factors that can help improve maternal and child health. Unfortunately, 47% of mothers in the United States typically exceed the amount of gestational weight gain recommended by the 2009 Institute of Medicine (IOM) guidelines (1). Of particular relevance are the recommended gestational weight ranges for women with pre-pregnancy overweight and obesity: 6.8–11.3 kg (15–25 lbs) for women with a body mass index (BMI) between 25 and 29.9 kg/m^2^ (overweight); and 5.0–9.1 kg (11–20 lbs) for women with a BMI of 30 kg/m^2^ or greater (obesity) (2). Excess weight gain during pregnancy is associated with negative short- and long-term health consequences for both mothers and their children including gestational diabetes mellitus, preeclampsia, postpartum weight retention, congenital abnormalities, stillbirth, and fetal macrosomia (3–7). Over 50% of women entering pregnancy can be classified as overweight or obese (8). These women are particularly vulnerable to excess gestational weight gain, with double the risk of gaining more than the recommended amount of weight during pregnancy compared to women who are of normal weight (9).

Pregnancy may be an opportune time for weight management since many women have regular contacts with health-care professionals and are potentially more motivated to maintain a healthy lifestyle (10, 11). However, there are several challenges to successful antenatal weight management. Barriers often cited by pregnant women revolve around pragmatic issues associated with attending a weight management program, including a lack of time to attend sessions due to competing demands and geographical constraints that may hinder the receipt of services (12, 13). Health-care providers also encounter significant barriers to providing weight management, such as lack of training in delivering weight counseling (14) and minimal time during appointments to discuss these issues (15). Results from interventions to prevent excessive gestational weight gain among women who have overweight or obesity have been modest. In a recent meta-analysis, pregnant women with overweight or obesity who were enrolled in lifestyle interventions gained an average of 1.7 kg less than control groups (16). Taking these challenges together, including the modest outcomes and logistical challenges documented thus far, low burden, effective, and scalable weight management interventions are needed for this population.

Technology-based interventions delivered in a woman’s home can help decrease the logistical constraints and burden common with weight management interventions (17, 18). Telemedicine is a technology-based approach that has become increasingly popular due to its high potential to be integrated into clinical practice and capacity to maintain patient-provider contact. It can include telephone counseling, text messages, web-based programs, and other wearable and WiFi devices that monitor weight and activity. In non-pregnant samples, telephone counseling produces similar weight loss compared to in-person counseling (19, 20). Of all the telehealth approaches, it seems that telephone counseling may provide the highest treatment intensity to optimize weight outcomes. Counseling visits can be combined with newer technologies such as “smart” scales to help ensure that important elements of weight-loss counseling are not omitted (21). These in-home body-weight scales transmit a patient’s weight to a clinician *via* WiFi. Health-care providers can then provide women with tailored feedback based on objective measures. “Smart” scales have been effective in obesity treatment interventions in non-pregnant samples (22), but the outcomes of this approach during pregnancy are not known. Little is known about the efficacy of this strategy in limiting excessive gestational weight gain.

The purpose of this pilot, randomized controlled trial was to test the feasibility and preliminary efficacy of an intervention consisting of telephone counseling focused on limiting excess weight gain during pregnancy plus WiFi weighing compared to a treatment as usual (TAU) control group. The primary aim was to compare the amount of weight gained during pregnancy among women in the intervention group as compared to women who received TAU. The hypothesis was that women in the intervention group would gain significantly less weight than those in the TAU. We also hypothesized that a smaller proportion of women in the intervention group would exceed the IOM’s recommended ranges for weight gain. An exploratory aim was to compare caloric intake among women in the intervention group as compared to women in the TAU group. We hypothesized that women in the intervention group would consume fewer total calories. Finally, we described the acceptability of the intervention and the usefulness of each of its components, as rated by participants.

## Materials and Methods

### Participants and Design

Women who were up to 16 weeks pregnant were recruited from two obstetrics clinics in Philadelphia, PA, USA. Inclusion criteria were self-reported pre-pregnancy BMI between 25 and 50 kg/m^2^; 18 and 40 years of age; and ability to read and understand English. Exclusion criteria were diabetes mellitus; history of gestational diabetes; twins or other multiples; HIV; and chronic steroid use. Women were screened at the clinics, self-reported pre-pregnancy height and weights were recorded, and pre-pregnancy BMI was calculated. Participants’ electronic medical records were accessed to obtain the mother’s weight at gestational age of 16 weeks (or weight closest to that age; range 13–16 weeks). The women provided written informed consent in accordance with the Declaration of Helsinki if they reached the inclusion criteria and were enrolled in the study after they completed a baseline questionnaire with contact and demographic information, and information about their eating habits and exercise routines. This study was reviewed and approved by the University of Pennsylvania Institutional Review Board.

### Intervention

The intervention consisted of weekly, 20-min telephone counseling sessions with a dietician between weeks 16 and 36 gestation or delivery of their baby, whichever came first. The dietician was trained in behavioral weight-loss treatment. A treatment manual based on the Look AHEAD trial was adapted from a behavioral weight-loss intervention to a behavioral weight management intervention focusing on appropriate gestational weight gain (23). The sessions addressed the domains associated with weight control: nutrition, exercise, and lifestyle modification strategies to improve adherence to the diet and activity plan, decrease disordered eating and response to environmental food cues, and improve mood and stress. Participants also kept food records to help them stay within recommended calorie ranges, aiming for an increase of only 300 cal per day above pre-pregnancy weight maintenance levels. As recommended in the IOM guidelines (2), weight gain goals during the second and third trimesters were 0.6 lbs/week for women with overweight and 0.5 lbs/week for women with obesity. These calculations assumed a 1.1- to 4.4-lb gain in the first trimester and were adjusted in each participant’s weight gain charts according to her actual first trimester gains. The women in this group were asked to weigh themselves weekly with a WiFi scale (www.Withings.com). The WiFi scales transmitted their weights to personalized weight charts that could be accessed *via* the Internet by the participant and study staff for remote weight monitoring and to give feedback to participants. During each weekly phone call, the study dietician reviewed a treatment session and gave individualized feedback based on the measured weights, verbal review of the food records, and any other adherence issues that the participants presented.

The TAU group received the counseling typically provided at their obstetrics visits on nutrition, exercise, and weight gain goals (24). They did not receive the telephone and WiFi weighing intervention, or specific feedback from study staff on the course of weight gain during their pregnancies. All women who were enrolled in the study completed monthly, 24-h food recalls using the Internet-based Automated Self-administered 24-h Recall (ASA24) (25).

### Measures

#### Demographic and Clinical Information

Participants completed a baseline survey that included questions on age, race, marital status, education, pregnancy history, and current smoking status.

#### Weight and Birth Outcomes

Pre-pregnancy weight was self-reported. For participants who had a weight recorded in the electronic medical record in the year prior to their pregnancy (TAU, *n* = 9 and intervention, *n* = 9), the mean difference between self-reported and measured weight was 1.8 ± 13.5 lbs. A structured medical abstraction form was used to extract data from participants’ electronic medical records. Final pregnancy weight was taken from the last measured weight during participants’ prenatal visits. Maternal and neonatal birth outcomes and maternal weight at 6 weeks postpartum, where available, were also extracted.

#### Questionnaires

Caloric intake was calculated from 24-h food records that were completed on the internet (25). Additional questionnaires were administered at baseline to provide a more comprehensive picture of these participants’ eating patterns, sleep duration, mood, and physical activity. Night eating was assessed using the Night Eating Questionnaire, a 14-item validated measure of night eating syndrome (NES) (26). All items, except one used to screen for the parasomnia sleep-related eating disorder, were scored on a 0 to 4 Likert scale and summed to obtain a global score. The NEQ has a possible range of 0–54 with higher scores indicating more NES symptoms; a score ≥25 is suggestive of NES. The Cronbach’s alpha in this sample was 0.66. Binge eating was measured using self-reported questions from the Eating Disorder Examination-Questionnaire (27). The items asked individuals if they consumed an unusually large amount of food while feeling a sense of loss control over eating during the past 28 days as well as the number of episodes over the past 4 weeks. We assessed average hours of sleep per night using the Pittsburgh Sleep Quality Index (28). Participants completed the 10-item Edinburgh Postnatal Depression Scale (EPDS) (29). Items were scored on a 0 to 3 Likert scale and summed to generate a total score. Higher scores indicated more depressive symptoms and scores of ≥10 indicate a moderate to high risk of perinatal of depression. The Cronbach’s alpha in this sample was 0.88. Stress was assessed using the 4-item Perceived Stress Scale (PSS) with a possible range of 0–15 and higher scores indicating more stress (30). Norms suggest a typical score is 6.4 (SD = 3.1) for women (31). The Cronbach’s alpha was 0.65. At baseline, participants were asked about the amount of time spent each week on physical activity (i.e., sports, leisure, or recreational activities) since becoming pregnant. At week 36, participants in the intervention group completed questions that assessed how helpful the intervention was to them. Scores ranged from 1 (did not help at all) to 5 (very helpful).

### Statistical Analysis

#### Power Analysis

The proposed study was powered to detect differences between groups for the primary aim. If the Intervention group limited weight gain to 7 kg (SD = 5.5), as it was in a previous study by Wolff et al. (32) and the TAU group gained 14.3 kg (SD = 9.9), as they did in the investigators’ previous study (33), then 21 participants per group would yield 80% power, using a two group Satterthwaite *t*-test with a 0.05 two-sided alpha level (nQuery Advisor, version 6). If the Intervention group gained 8 kg, then 28 participants per group would yield 80% power. Given the short time-constraints of the study, we proposed to recruit 21 participants per group.

Data were analyzed using SPSS version 24.0. Descriptive statistics were used to characterize the sample (i.e., means, SDs, percentages). Independent sample *t*-tests and chi-square tests were conducted to compare the intervention and TAU groups on all main outcome measures. Pearson correlations were used to assess the relationship between intervention session attendance and weight loss. We modeled caloric intake over the intervention period using linear mixed models. A *p*-value < 0.05 was considered statistically significant.

## Results

**Figure 1:**
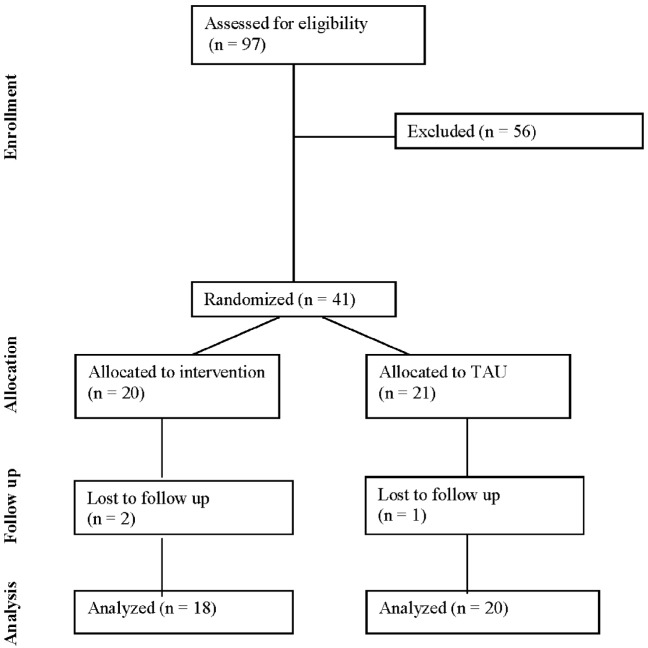
CONSORT diagram.

The CONSORT diagram for this study is presented in Figure 1. In the total sample (*n* = 41), participants had a mean age of 28.7 ± 5.8 years and the sample was 53.7% black, 39.0% white, 7.2% other. Approximately half (51.2%) of participants were married and 48.8% had graduated from college. One participant was a current smoker. Mean gravidity (i.e., the number of times a woman has been pregnant) was 1.3 ± 1.5 and parity (i.e., the number of times a woman has given birth to a fetus of 24 weeks or more) was 0.8 ± 1.0. Participants had a mean pregestational BMI of 31.1 ± 6.2 kg/m^2^ with 53.7% of participants beginning the pregnancy in the overweight range and 46.3% reporting a pregestational BMI in the obese range. On average, scores on the EPDS were 6.6 ± 5.6 with 10 (24.4%) women at moderate to high risk of perinatal depression. The mean PSS score was 5.7 ± 3.8, which was within a normal range, and NEQ score was 14.5 ± 6.7, suggesting the presence of subthreshold night eating symptoms. Participants reported an average of 7.1 ± 1.5 h of sleep per night. One participant reported binge eating (two episodes in the past 4 weeks) at baseline. Participants reported engaging in an average of 46.5 ± 91.8 min of physical activity per week with 68.3% reporting no weekly physical activity. Baseline characteristics were not significantly different between the intervention (*n* = 20) and treatment (*n* = 21) groups (*p*s > 0.05; Table 1).

**Table 1:** Baseline demographic and clinical characteristics by treatment arm.

	Intervention (*n* = 20) mean (SD) or *N* (%)	TAU (*n* = 21) mean (SD) or *N* (%)	*p*-Value
Age, years	29.1 (6.4)	28.4 (5.2)	0.73
Race			
Black	9 (45.0)	13 (61.9)	0.36
White	10 (50.0)	6 (28.6)	
Other	1 (5.0)	2 (9.5)	
Married	12 (60.0)	9 (42.9)	0.35
Education			
Not college graduate	10 (50.0)	11 (52.4)	0.88
College graduate	10 (50.0)	10 (47.6)	
Gravidity	1.0 (1.1)	1.7 (1.7)	0.11
Parity	0.8 (1.0)	0.9 (1.1)	0.63
Pregestational BMI (kg/m^2^)	31.0 (4.8)	31.3 (7.5)	0.90
Pregestational BMI status			
Overweight	10 (50.0)	12 (57.1)	0.76
Obese	10 (50.0)	9 (42.9)	
Depressive symptoms (EPDS)	6.9 (4.4)	6.3 (6.6)	0.75
Perceived stress scale (PSS)	6.2 (2.9)	5.1 (4.5)	0.37
Night eating (NEQ)	15.4 (6.5)	13.6 (7.0)	0.40
Sleep per night, hours (PSQI)	7.3 (1.7)	6.9 (1.2)	0.36

*TAU, treatment as usual; EPDS, Edinburgh Postnatal Depression Scale; PSS, Perceived Stress Scale; NEQ, Night Eating Questionnaire; PSQI, Pittsburgh Sleep Quality Index; BMI, body mass index*.

Before the intervention began, the amount of weight gain in the first 16 weeks was 4.8 ± 4.6 kg in the intervention group and 3.0 ± 5.5 kg (*p* = 0.33) in the TAU group. Total gestational weight gain did not differ significantly (*p* = 0.29) between the intervention (15.5 ± 5.3 kg; range: 4.5–25.4 kg) and TAU (13.3 ± 6.8 kg; range: −1.8 to 28.6 kg) groups. The Cohen’s *d* for gestational weight gain was 0.36. The proportion of the sample exceeding IOM weight gain recommendations was high in both the intervention (83.3%) and TAU (70%, *p* = 0.74) groups. The intervention and TAU groups did not differ in change in reported caloric intake over the course of the intervention (2,154 ± 251.3 vs. 1,972.0 ± 202.7 kcals; *p* = 0.58).

Participants in the telemedicine group completed a mean of 12.5 ± 6.4 telephone counseling sessions. The number of intervention sessions completed inversely correlated with pre-pregnancy BMI (*r* = −0.46, *p* = 0.04) and positively with gestational weight gain (*r* = 0.55, *p* = 0.02). Internet access varied throughout the study, with 58% having access across the entire length of the intervention. Of the 14 intervention participants who completed the week 36 assessment, most participants rated the intervention components as helpful in affecting pregnancy weight gain (Table 2). Participants rated talking with the dietician as most helpful (4.4 ± 0.9), followed by weekly weighing on the scale (4.3 ± 0.8) and sharing their weight each week with the study staff (4.1 ± 0.8).

**Table 2:** Intervention process rating scores (*n* = 14).

	Mean (SD)
Stepping on scale each week	4.29 (0.83)
Sharing your weight each week with study staff	4.07 (0.83)
Talking with the dietician each week	4.36 (0.93)
Using materials in the workbook	3.50 (1.35)
Conversing with your obstetrician and other medical care providers	2.36 (1.39)
Keeping daily, paper food logs	4.00 (0.96)
Completing internet-based 24-h food recalls^a^	2.56 (1.32)

*Scores range from 1 (did not help at all) to 5 (very helpful) in affecting pregnancy weight gain*.

*^a^Nine participants completed this question*.

Sixteen participants in the intervention group and 16 in the TAU group had data available on weight change from pre-pregnancy to 6-week postpartum, which did not differ between groups (4.8 ± 4.6 vs. 3.0 ± 5.5 kg, respectively, *p* = 0.33). The intervention and TAU group did not differ in their 1-h 50 g glucose test results (116.0 ± 35.3 vs. 113.9 ± 23.2 mg/dL, *p* = 0.82). Gestational week at delivery did not differ between the intervention (38.6 ± 1.8) and TAU groups (39.4 ± 1.3; *p* = 0.10). The intervention and TAU groups did not differ in the birth weight (3,254.6 ± 653.1 vs. 3,381.1 ± 552.9 g, *p* = 0.52) or 5-min Apgar scores (8.8 ± 0.7 vs. 8.9 ± 0.5, *p* = 0.73) of their babies.

## Discussion

In this preliminary efficacy study, a telephone-based approach combined with WiFi weighings was not able to decrease excess gestational weight gain among this diverse sample of women with overweight or obesity. The majority of the sample gained more than the 2009 IOM guidelines for recommended weight gain, and birth outcomes did not differ between groups. A small number of studies have tested the efficacy of technology-based interventions among samples of pregnant women with overweight or obesity. Some studies have shown that technology-based interventions can result in minimal to modest benefits in gestational weight gain (17, 18). However, this technology-based intervention yielded no benefits for limiting excessive gestational weight gain as compared to TAU. Previous weight management interventions have been more successful in controlling weight gain among women of normal weight, but not women with overweight or obesity [see Herring et al. (34) for review]. Some factors may be the truncated recommended weight gain ranges for these groups, reduced activity levels, and/or poorer diet quality.

In both groups, there was clinically significant weight gain before the intervention started at 16 weeks. Many women gain much of their pregnancy weight during the first trimester (35). Early gestational weight gain is also predictive of subsequent weight gain (36). With the IOM’s recommended upper limit of 11.3 kg (25 lbs) for women with overweight and 9.1 kg (20 lbs) for women with obesity, our intervention group was about halfway to this mark by 16 weeks. The 1.8 kg (4 lbs) difference between the intervention and TAU groups at week 16 seemed to follow the groups at delivery and at 6 weeks postpartum. Therefore, it is likely that interventions focused on preventing excessive gestational weight gain should start earlier, and ideally before conception, as recommended by the IOM (2). The strategy of treating overweight/obesity with lifestyle modification prior to conception has been successful in improving outcomes among women with polycystic ovarian syndrome (PCOS), a common endocrine condition affecting women of reproductive age (37, 38). Future studies are necessary to assess the efficacy of this approach on maternal and neonatal outcomes in women with overweight/obesity without PCOS.

In this study, telephone sessions were a feasible way to deliver weight-loss counseling among pregnant women. The WiFi scales relied on cellular service and a significant number of participants did not have reliable WiFi service to use such scales. Not having access to reliable and high-speed internet could reduce feasibility and pose barriers when implementing these technologies. In the current study, participants self-reported the weight on their Withings scales to the dietician, and the weights were entered manually on the participants’ Internet-based weight charts.

It is possible that the WiFi weighing and lifestyle counseling telephone sessions could improve outcomes if combined with other methods for weight control. For example, telephone counseling sessions have been most frequently used with in-person visits and have been an effective strategy in some (39, 40), but not all studies (41–43). Several other technologies have been used to address gestational weight gain with varying success including websites, mobile phone applications, text messaging, email, and video (18). A recent study combined several of these approaches to decrease postpartum weight retention among women with overweight/obesity recruited through the Women, Infant, and Children program (44). Women in the intervention were significantly more likely to return to pre-gravid weight at 6 months postpartum, as compared to the usual care group. Another potential direction is integrating technology to improve self-monitoring and to allow for more intensive monitoring by healthcare providers. Self-monitoring of weight, eating behaviors, and physical activity is a crucial part of weight loss as well as maintenance. WiFi scales were provided as part of the intervention to help patients monitor their weight as well as to allow interventionists to provide feedback to patients remotely. Participants viewed the scales as helpful. Incorporating other technologies to improve self-monitoring of food intake (e.g., food monitoring apps) and physical activity (e.g., wearable activity trackers) may help to promote better outcomes. Evidence is currently inconclusive in technology-based interventions for weight management during pregnancy, and more research is needed to determine the ideal treatment components, delivery, and intensity.

Limitations to this study include a small sample size with limited statistical power, use of self-reported pregestational weight, and short-term follow-up. We relied on self-reported pre-pregnancy weights which can lead to inaccurate weight classification. Using the subset of participants with available pre-pregnancy weights, we found that the mean difference was less than 0.8 kg (1.8 lbs), but that variability was wide. In addition, physical activity was not tracked or monitored in this study. Use of 24-h recalls could have resulted in some reactivity among the women in the TAU group. However, this method of dietary assessment is considered to be one of the least biased among self-report methods in terms of reactivity (45).

In conclusion, an intervention that included behavioral nutrition counseling *via* phone combined with WiFi measures of weight was not effective in reducing excess gestational weight gain among overweight and obese pregnant women. We hypothesize that starting an intervention program earlier or having a more intensive program might yield a better impact overall. Future research is needed to determine the optimal time to start such an intervention, as well as the components and method of delivery.

## Ethics Statement

The women provided written informed consent in accordance with the Declaration of Helsinki if they reached the inclusion criteria and were enrolled in the study after they completed a baseline questionnaire with contact and demographic information and information about their eating habits and exercise routines. This study was reviewed and approved by the University of Pennsylvania Institutional Review Board.

## Author Contributions

KA, SSrinivas, and DS made substantial contributions to the conception or design of the work; AC, SSrinivas, SStudt, LD, DS, and KA contributed to the acquisition, analysis, or interpretation of data for the work. All authors participated in the drafting the work or revising it critically for important intellectual content, final approval of the version to be published, and agreement to be accountable for all aspects of the work in ensuring that questions related to the accuracy or integrity of any part of the work are appropriately investigated and resolved.

## Conflict of Interest Statement

The authors declare that the research was conducted in the absence of any commercial or financial relationships that could be construed as a potential conflict of interest.
